# Everolimus regulates the activity of gemcitabine-resistant pancreatic cancer cells by targeting the Warburg effect via PI3K/AKT/mTOR signaling

**DOI:** 10.1186/s10020-021-00300-8

**Published:** 2021-04-13

**Authors:** Jing Cui, Yao Guo, Heshui Wu, Jiongxin Xiong, Tao Peng

**Affiliations:** Department of Pancreatic Surgery, Union Hospital, Tongji Medical College, Huazhong University of Science and Technology, Wuhan, China

**Keywords:** Pancreatic cancer, Gemcitabine, Everolimus, Drug resistance, Metabolism

## Abstract

**Background:**

Gemcitabine (GEM) resistance remains a significant clinical challenge in pancreatic cancer treatment. Here, we investigated the therapeutic utility of everolimus (Evr), an inhibitor of mammalian target of rapamycin (mTOR), in targeting the Warburg effect to overcome GEM resistance in pancreatic cancer.

**Methods:**

The effect of Evr and/or mTOR overexpression or GEM on cell viability, migration, apoptosis, and glucose metabolism (Warburg effect) was evaluated in GEM-sensitive (GEM_sen_) and GEM-resistant (GEM_res_) pancreatic cancer cells.

**Results:**

We demonstrated that the upregulation of mTOR enhanced cell viability and favored the Warburg effect in pancreatic cancer cells via the regulation of PI3K/AKT/mTOR signaling. However, this effect was counteracted by Evr, which inhibited aerobic glycolysis by reducing the levels of glucose, lactic acid, and adenosine triphosphate and suppressing the expression of glucose transporter 1, lactate dehydrogenase-B, hexokinase 2, and pyruvate kinase M2 in GEM_sen_ and GEM_res_ cells. Evr also promoted apoptosis by upregulating the pro-apoptotic proteins Bax and cytochrome-c and downregulating the anti-apoptotic protein Bcl-2. GEM was minimally effective in suppressing GEM_res_ cell activity, but the therapeutic effectiveness of Evr against pancreatic cancer growth was greater in GEM_res_ cells than that in GEM_sen_ cells. In vivo studies confirmed that while GEM failed to inhibit the progression of GEM_res_ tumors, Evr significantly decreased the volume of GEM_res_ tumors while suppressing tumor cell proliferation and enhancing tumor apoptosis in the presence of GEM.

**Conclusions:**

Evr treatment may be a promising strategy to target the growth and activity of GEM-resistant pancreatic cancer cells by regulating glucose metabolism via inactivation of PI3K/AKT/mTOR signaling.

**Supplementary Information:**

The online version contains supplementary material available at 10.1186/s10020-021-00300-8.

## Background

Pancreatic cancer is a malignant tumor of the digestive system with late clinical detection and poor prognosis. Epidemiological data has shown that the five-year survival rate of pancreatic cancer is below 8% and exhibits an upward trend (Wang 2018). Although surgical resection is the main technique of treating pancreatic cancer, prognosis remains poor even after curative surgery because of the high recurrence rate. Gemcitabine (GEM) is currently the main drug applied in the clinical treatment of pancreatic cancer. Adjuvant chemotherapy with GEM improved the overall survival of patients with macroscopically resected pancreatic cancer (Oettle 2013). However, a major issue related to GEM use is the development of drug resistance in cancer cells (Voutsadakis 2011). Multiple signaling pathways and downstream proteins such as phosphatidylinositol-4,5-bisphosphate 3-kinase/protein kinase B/mammalian target of rapamycin (PI3K/AKT/mTOR), nuclear factor-κB, and mitogen-activated protein kinases are involved in GEM resistance in pancreatic cancer. The regulation of these pathways results in changes in drug pumping, drug intake, target molecules, and drug inactivation in tumor cells (Wang et al. 2011; Jin 2017; Wang 2017). Given the wide applicability of GEM across all stages of pancreatic cancer, the development of strategies to reverse or prevent GEM resistance continues to be an urgent need.

Abnormal cell metabolism is one of the hallmarks of cancer (Hanahan and Weinberg 2011) and is reflected by altered glucose metabolism. Tumor cells produce a large amount of lactic acid through aerobic glycolysis and reduced mitochondrial oxidation phosphorylation, a phenomenon known as the “Warburg effect” (Epstein et al. 2017). Excessive lactic acid production creates an acidic tumor microenvironment, which promotes the migration and invasion of tumor cells (Hirschhaeuser et al. 2011). In addition to promoting tumorigenesis, the metabolic characteristics of cancer cells provide an environment that often favors drug resistance. Glucose transporter (GLUT) inhibition can enhance the sensitivity of pancreatic cancer cells to GEM via the inhibition of glucose uptake (Jiang 2017). In our preliminary study, we showed that glucose uptake and lactic acid formation were enhanced in GEM-resistant pancreatic cancer cell lines. However, the specific mechanism underlying the Warburg effect in pancreatic cancer resistance to GEM is still unclear.

A number of oncogenes and pathways have been identified to be involved in glucose metabolism, suggesting their potential role in tumorigenesis. The PI3K/AKT/mTOR pathway is abnormally activated in a variety of tumor cells, regulating tumor occurrence, development, drug resistance, and the Warburg effect (Jiang 2017; Shen et al. 2017). PI3K/AKT/mTOR activation has been reported to trigger hypoxia-inducible factors, contributing to tumor progression and survival by regulating the transcription of oncogenes and GLUTs (Rohwer and Cramer 2011; Pore 2006). In addition, the PI3K/AKT/mTOR signaling pathway positively regulates the Warburg effect by upregulating the expression of GLUTs and lactate dehydrogenase (LDH) and stimulating glutaminolysis in tumor cells (Courtnay 2015; Bao et al. 2015; Guo 2016). During glycolysis, a large amount of deoxycytidine triphosphate (dCTP) is produced, promoting DNA repair and inducing the expression of glycolytic enzymes. However, GEM absorbed by nucleoside transporters can be metabolized by deoxycytidine kinase into active GEM monophosphate and GEM diphosphate, which embed into the DNA double helix to inhibit DNA repair. During tumor cell metabolism, dCTP is produced at high concentrations and competitively binds to DNA, thereby reducing the sensitivity of pancreatic cancer cells to GEM and lowering its therapeutic effect (Shukla 2017).

Everolimus (Evr) is an inhibitor of mTOR serine/threonine kinase that acts as an immunosuppressor in organ transplantation (Buti et al. 2016). In recent years, the potential of Evr as an anti-tumor drug has been increasingly highlighted. Evr has been shown to promote the apoptosis of GEM-resistant (GEM_res_) pancreatic cancer cells by activating caspase-3 and caspase-7 through PI3K/AKT/mTOR signaling (Peng and Dou 2017). In addition, PI3K/AKT reportedly promoted abnormal fibroblast proliferation by upregulating the expression of metabolism-related enzymes and triggering the Warburg effect, but this effect was reversed by Evr (Parker et al. 2015). We previously demonstrated that Evr significantly reduced glucose uptake and lactic acid formation in GEM-sensitive (GEM_sen_) and GEM_res_ pancreatic cancer cells, and the inhibition efficiency of Evr on GEM_res_ cells was significantly higher than that on GEM_sen_ cells. By inhibiting mTOR, Evr affects tumor cell metabolism and regulates tumor cell resistance, but its mechanism of action needs to be further investigated.

The aim of the present study was to explore the effect of Evr on the activity of GEM_res_ pancreatic cancer cells, including cell migration, apoptosis, glucose metabolism, and molecular signaling. The effects of Evr and GEM were validated in an in vivo model of pancreatic tumor progression. The potential molecular mechanisms associated with the therapeutic action of Evr and whether Evr has the ability to enhance the sensitivity of GEM_res_ cells to GEM were investigated and discussed.

## Methods

### Cell culture, transfection, and drug treatment

GEM_sen_ and GEM_res_ BxPC-3 human pancreatic cancer cells were provided by Dr. Yiwei Li (Karmanos Cancer Institute, Wayne State University, Detroit, MI). Cells were cultured in Dulbecco’s modified Eagle’s medium (Gibco; Thermo Fisher Scientific, Waltham, MA) supplemented with 5% fetal bovine serum (FBS), 100 U/mL penicillin, and 100 mg/mL streptomycin at 37 °C in humidified air with 5% CO_2_. Cells were harvested by trypsinization and washed with phosphate-buffered saline (PBS). For transfection, pCDH-CMV-MCS-EF1-CopGFP-T2A-Puro vectors were purchased from Addgene (Watertown, MA). mTOR mRNA was amplified through polymerase chain reaction (primary sequence: forward, 5′-CTCTAGATGCTTGGAACCGGACCTGCC-3′; reverse, 5′-GGAATTCTTACCAGAAAGGGCACCAGCC-3′) and inserted into the vector with *EcoRI* and *BamHI* restriction sites to produce mTOR overexpression (ov-mTOR) vectors. GEM_sen_ and GEM_res_ cells were transfected with ov-mTOR vectors or the corresponding empty vector (EV, negative control) using Lipofectamine 2000 (11668-027, Invitrogen, Waltham, MA) for 48 h according to the manufacturer’s instruction. Drug administration was performed by adding 0.1 μM GEM (G127944, Aladdin, Shanghai, China) or/and 30 mM Evr (E125341, Aladdin) to GEM_sen_ and GEM_res_ cells, individually or in combination for 48 h.

### Quantitative reverse transcription polymerase chain reaction (qRT-PCR)

RNA was extracted using TRIzol (15596026, Ambion, Inc., Foster City, CA) and reverse-transcribed into cDNA using the PrimeScript II RTase kit (Takara, Kyoto, Japan). qRT-PCR was performed using the SYBR Green PCR kit (KM4101, KAPA Biosystems, Wilmington, MA) on a CFX Connect 96 apparatus (Bio-Rad, Hercules, CA) with the following primer sequences: mTOR forward, 5′-CGGTGTTGCAGAGACTTGATGGAG-3′ and reverse, 5′-CTGTGAAGGCAGAAGGTCGGAATG-3′; GAPDH forward, 5′-CCACTCCTCCACCTTTG-3′ and reverse, 5′-CACCACCCTGTTGCTGT-3′. The experimental conditions were as follows: initial denaturation at 95 °C for 3 min; 39 cycles of denaturation at 95 °C for 5 s, annealing at 56 °C for 10 s, and extension at 72 °C for 25 s; and final extension at 65 °C for 5 s and 95 °C for 50 s. Relative fold change in mRNA expression was analyzed with the 2^−ΔΔCt^ method.

### 3-(4,5-Dimethylthiazol-2-yl)-2,5-diphenyltetrazolium bromide (MTT) assay

The viability of cancer cells was detected by a colorimetric MTT method. Cells were seeded in 96-well plates at 1 × 10^4^ cells per well. MTT reagent (20 µL) was added to each well after treatment for 48 h and incubated for 2–4 h at 37 °C. After the purple precipitate became visible, the medium was removed and 150 µL of dimethyl sulfoxide was added to each well. After 10 min of low-speed shaking, the absorbance of the wells was recorded at 570 nm.

### Biochemical evaluation of glucose uptake, lactic acid production, and adenosine triphosphate (ATP) level

Glucose uptake was evaluated by measuring the uptake of 2-deoxyglucose (2-DG) following the protocols of an assay kit (KA4086, Abnova, Taipei, Taiwan). All required reagents are provided within the kit. Briefly, non-transfected or ov-mTOR-transfected cells were seeded in a 96-well plate for 6 h at 37 °C in 5% CO_2_. The medium was aspirated and the cells were washed twice with KRPH buffer. Then, 90 μL of glucose uptake buffer was added to each well and incubated for 1 h, after which 10 μL of 2-DG solution was added to each well and incubated for 30 min. The cells were washed and lysed using 25 μL of acidic lysis buffer per well. Then, 50 μL of 2-DG uptake assay working solution was added to each well and the plate was incubated at room temperature for 1 h. Glucose uptake was evaluated by measuring the absorbance of the wells at 570/610 nm using a microplate reader. The levels of lactic acid (A019-2) and ATP (A095) were evaluated using corresponding assay kits from Nanjing Jiancheng Bioengineering Institute (Nanjing, China). Cells were resuspended at 1 × 10^6^ cells/mL and centrifuged at 400×*g* at 4 °C for 5 min and the supernatant was discarded. The cells were resuspended in 200 μL of PBS and lysed using an ultrasonication apparatus at a frequency of 30 kHz. After lysis, the samples were centrifuged at 400×*g* at 4 °C for 5 min, and the supernatant was collected for analysis. To examine lactic acid content, the samples were mixed with enzyme solutions provided in the assay kit at ratios specified in the protocol. After 10 min of reaction at 37 °C in a water bath, a stop solution was added and the absorbance was measured at 530 nm. To evaluate ATP content, the samples were mixed with reagents provided in the assay kit at ratios specified in the protocol and incubated for 30 min at 37 °C in a water bath. A precipitant was added to the sample and after mixing, the sample was centrifuged at 4000 rpm for 5 min. Then, 300 μL of supernatant was collected and mixed with 500 μL of color reaction mixture. After 2 min of incubation at room temperature, a stop solution was added and the absorbance was measured at 636 nm.

### Western blot

A total of 30 μg of protein was resuspended in sodium dodecyl sulfate (SDS) sample buffer and boiled for 5 min. Equal amounts of total protein were loaded onto a 10% SDS-containing polyacrylamide gel and separated by SDS-polyacrylamide gel electrophoresis (Sigma-Aldrich, St. Louis, MO), followed by electrotransfer to polyvinylidene difluoride membranes (Sigma-Aldrich). The membranes were saturated with blocking buffer for 1 h at room temperature and incubated with primary antibodies against GLUT1 (ab652, 1:1000, abcam, Cambridge, UK), lactate dehydrogenase-B (LDHB, 1:2000, PAB35369, Bioswamp, Wuhan, China), hexokinase 2 (HK2, 1:2000, PAB30271, Bioswamp), pyruvate kinase isozymes M2 (PKM2, 1:2000, PAB31790, Bioswamp), Bax (1:2000, PAB30040, Bioswamp), Bcl-2 (1:2000, PAB30041, Bioswamp), cytochrome-c (Cyt-c, 1:1000, ab90529, abcam), p-AKT (1:500, ab8933, abcam), p-PI3K (1:1000, ab182651, abcam), phosphorylated mTOR at serine 2481 (mTOR p-S2481, 1:1000, ab137133, abcam), mTOR p-S2448 (1:1000, ab84400, abcam), p-P70S6K (1:1000, ab59208, abcam), multi-drug resistance protein 1 (MDR1, 1:2000, PAB30805, Bioswamp), multi-drug resistance-associated protein 1 (MRP1, 1:1000, PAB33537, Bioswamp), breast cancer resistance protein (BCRP, 1:10,000, ab108312, abcam), or GAPDH (1:2000, PAB36264, Bioswamp; or 1:5000, 10494-1-AP, Proteintech) at 4 °C overnight. Then, the membranes were washed with Tris-buffered saline and incubated in goat anti-rabbit secondary antibody (1:20,000, PAB160011, Bioswamp) for 2 h at room temperature. Immunoreactivity was visualized by colorimetric reaction using an enhanced chemiluminescence substrate buffer (Millipore, Burlington, MA). The membranes were scanned with Gel Doz EZ imager (Bio-Rad).

### Transwell migration assay

Prior to the experiment, cells were serum-starved for 24 h, and 24-well plates and Transwell inserts were washed with PBS for 5 min. The cells were trypsinized, washed with serum-free medium, and resuspended in medium containing 1% FBS at 1 × 10^5^ cells/mL. Then, 0.5 mL of cells were seeded in the top chamber of each Transwell insert, while 0.75 mL of medium containing 10% FBS was added to the bottom chambers. The well plate was incubated at 37 °C for 48 h and the medium was removed. The cells were washed with PBS and 1 mL of 4% paraformaldehyde was added to each well to fix the cells for 20 min. The fixative solution was removed and the cells were washed with PBS, after which 1 mL of 0.5% crystal violet solution was added to each well. After 30 min, the cells were washed three times with PBS and dried. Unattached cells were removed using a cotton swab and the migrated cells were visualized at × 200 under an optical microscope.

### Flow cytometry of cell apoptosis

Cell apoptosis was measured using an annexin V-phycoerythrin (PE)/7-aminoactinomycin D (7-AAD) flow cytometry kit (559763, BD Bioscience, Franklin Lakes, NJ) according to the manufacturer’s instructions. Cells were washed with ice-cold PBS three times and resuspended in binding buffer at a concentration of 1 × 10^6^ cells/mL. The cells were centrifuged at 400×*g* at 4 °C for 5 min and the supernatant was discarded. Then, the cells were resuspended in 1 mL of PBS and centrifuged again at 400×*g* at 4 °C for 5 min, and the supernatant was discarded. The cells were resuspended in 200 μL of PBS, after which annexin V-PE and 7-AAD dye solutions (5 μL of each) were added to the cells. After 30 min of incubation at 4 °C in the dark, 300 μL of PBS was added and the cells were analyzed by flow cytometry (Cytomics FC 500, Beckman Coulter, Brea, CA) within 1 h.

### Tube formation assay

Treated cells were resuspended in medium containing 10% FBS at 2 × 10^5^ cells/mL. In a pre-cooled 96-well plate, 50 μL of Matrigel was added to each well and the plate was incubated at 37 °C for 45 min. Then, 50 μL of cell suspension was added to each well and the well plate was incubated at 37 °C for 4 h, after which tube formation was visualized on the Matrigel.

### In vivo xenografted pancreatic tumor model

The study protocol was approved by the Institutional Review Board of Wuhan Myhalic Biotechnology Co., Ltd. (approval number HLK-20181102-01) and adhered to the “Guidelines for Animal Care and Use of the Model Animal Research Institute at Wuhan Myhalic Biotechnology Co., Ltd.” Six-week-old BALB/C nude mice (n = 48, male, 20–22 g, specific-pathogen-free) were obtained from Huafukang and housed at 22–26 °C and 50–60% humidity. The mice were divided into eight groups (n = 6 mice per group): transplantation with GEM_sen_ or GEM_res_ cells and one of four types of treatments (no drug, GEM, Evr, and GEM + Evr). After seven days of adaptive feeding, 0.1 mL of GEM_sen_ or GEM_res_ cells were injected subcutaneously at 1 × 10^7^ cells/mL for each mouse. Drug treatment began after one week. GEM or/and Evr was administered through tail vein injection at 40 mg/kg or/and 5 mg/kg, respectively, three times a week for three continuous weeks. Control mice (no drug) were administered an equal amount of physiological saline via tail vein injection. At the end of the 28-day experimental period, the mice were sacrificed with an overdose of sodium pentobarbital and tumor tissues were extracted for analysis.

### Ki67 and terminal deoxynucleotidyl transferase dUTP nick end labeling (TUNEL) staining

Tumor tissues were cut into small pieces and embedded in paraffin wax. The tissue samples were cut into 2-μm sections and transferred to glass slides. The sections were heated at 65 °C for 1 h and immersed twice in xylene for 15 min each. Then, the sections were washed with a graded concentration series of ethanol and then with running water for 10 min. For Ki67 staining, antigen retrieval was performed with 0.01 M sodium citrate buffer for 15 min and peroxidase activity was blocked with 4% H_2_O_2_ for 3 min in a humidified environment. After three washes with PBS, the sections were blocked with 0.5% bovine serum albumin for 10 min and washed three times with PBS. The sections were then incubated with Ki67 primary antibodies for 2 h at 37 °C and washed three times with PBS. Then, the sections were incubated with MaxVision secondary antibodies at room temperature for 30 min and washed three times with PBS. Color reaction was performed using diaminobenzidine and when color was detected, the sections were washed with tap water. Hematoxylin staining was performed for 3 min and the sections were washed for 10 min with tap water. After ethanol washing, the sections were washed with xylene and sealed. For TUNEL staining, the sections were immersed in proteinase K solution for 15 min at 37 °C and washed twice in PBS. Then the sections were incubated with TUNEL solution in a humidified environment in the dark for 1 h at 37 °C. The sections were washed three times with PBS and 50 μL of POD was added for 30 min at 37 °C. The sections were again washed three times with PBS and 50 μL of diaminobenzidine was added for 10 min at room temperature. After three washes in PBS, the sections were counterstained with hematoxylin, dehydrated, and transparentized. The sections were sealed and observed under a microscope. The relative amount of Ki67-positive and TUNEL-positive staining was evaluated using ImagePro Plus and expressed as the mean integrated optical density of brown staining.

### Statistical analysis

Data are expressed as mean ± standard deviation (SD) of three (n = 3, in vitro) or six (n = 6, in vivo) replicates from independent experiments. One-way analysis of variance with post hoc test was performed to compare differences between multiple groups using SPSS 19.0 software (IBM Corp., Armonk, NY). P < 0.05 was considered statistically significant.

## Results

### Effect of mTOR overexpression and Evr treatment on Warburg effect in GEM_sen_ and GEM_res_ cells

**Fig. 1:**
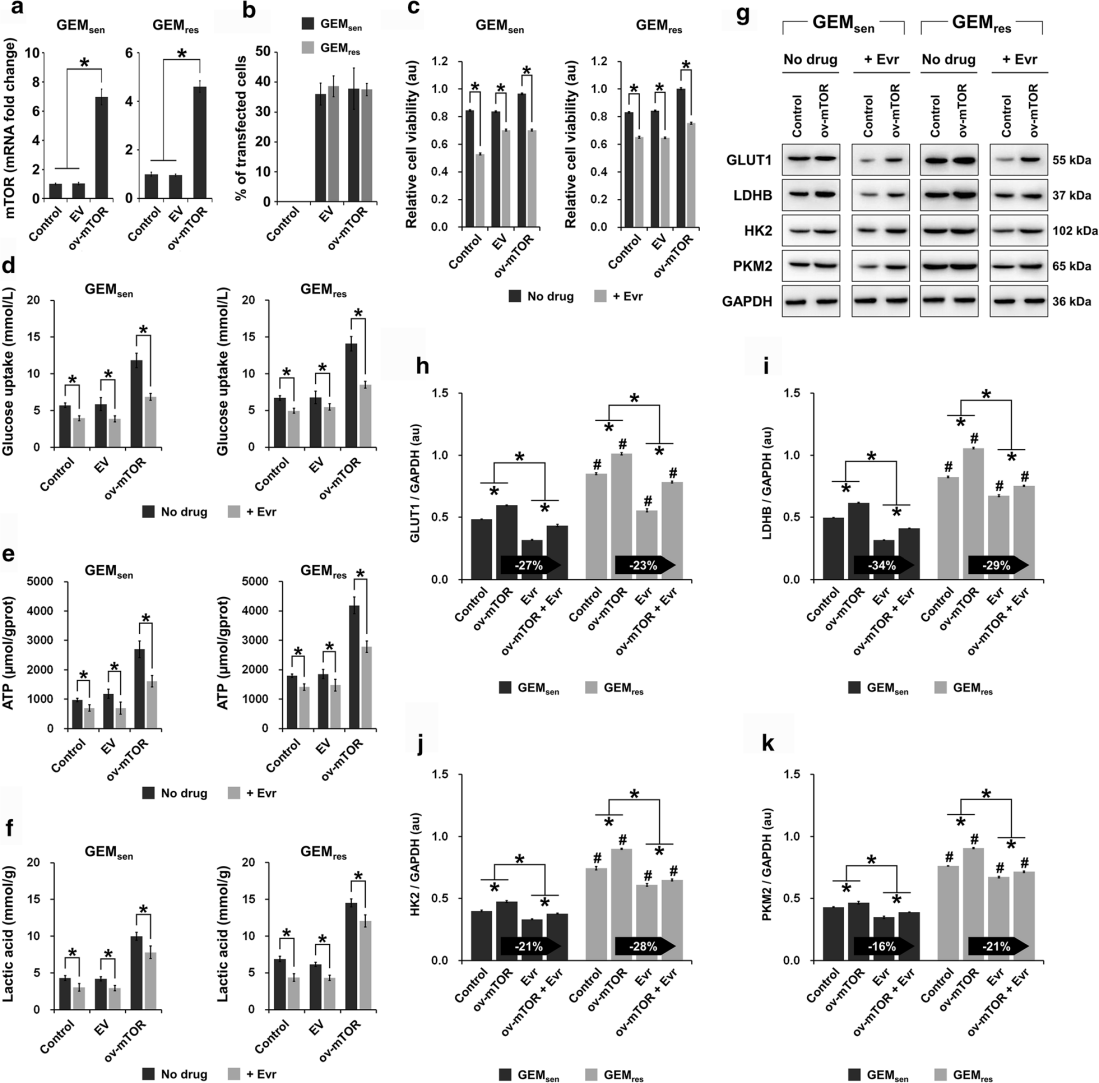
Effect of mTOR overexpression and Evr treatment on the Warburg effect in GEM_sen_ and GEM_res_ cells. The transfection efficiency of mTOR overexpression (ov-mTOR) vectors was evaluated by measuring **a** the relative mRNA expression of mTOR and **b** the percentage of positive fluorescence signal (transfected cells) in GEM_sen_ and GEM_res_ cells. The transfection efficiency was determined to be approximately 37%. **c** Effect of ov-mTOR on the viability of GEM_sen_ and GEM_res_ cells, with or without Evr treatment. **d** Glucose uptake, **e** ATP production, and **f** lactic acid generation in GEM_sen_ and GEM_res_ cells with or without ov-mTOR transfection or/and Evr treatment. mTOR overexpression promoted glucose uptake, ATP production, and lactic acid generation in both cell types, but its effect appeared to be less pronounced in GEM_res_ cells than that in GEM_sen_ cells. **g** Western blot of proteins associated with the Warburg effect. Quantification of the protein expression of **h** GLUT1, **i** LDHB, **j** HK2, and **k** PKM2 in GEM_sen_ and GEM_res_ cells with or without ov-mTOR transfection or/and Evr treatment, normalized to that of GAPDH as an internal control. From **h** to **k**, the numbers in the arrows represent the difference (increase or decrease) in protein expression between ov-mTOR and ov-mTOR + Evr. In ov-mTOR-transfected GEM_res_ cells, the suppressive effect of Evr on the expression of GLUT1 and LDHB was weaker than that in ov-mTOR-transfected GEM_sen_ cells. Conversely, the suppressive effect of Evr on the expression of HK2 and PKM2 was stronger in ov-mTOR-transfected GEM_res_ cells than that in ov-mTOR-transfected GEM_sen_ cells. The data are expressed as the mean ± standard deviation of three replicates (n = 3). *P < 0.05; ^#^P < 0.05 compared with the same treatment in GEM_sen_ cells. GEM: gemcitabine, Evr: everolimus; EV: empty vector (negative control); GEM_sen_: GEM-sensitive pancreatic cancer cells; GEM_res_: GEM-resistant pancreatic cancer cells; au: arbitrary units

Among several mTOR overexpression vectors that were examined in preliminary experiments, we selected the one that showed the highest efficiency in upregulating the expression of mTOR (data not shown). We first showed that mTOR overexpression (ov-mTOR) in both GEM_sen_ and GEM_res_ cells significantly elevated the mRNA expression of mTOR, as anticipated after transfection (Fig. 1a). Upon 48 h of transfection, GEM_sen_ and GEM_res_ cells were observed by bright-field and fluorescence microscopy (Additional file 1: Supplementary Figure S1). In the fluorescence images, cells transfected with fluorescent vectors are shown expressing positive green staining. The transfection efficiency of the ov-mTOR vectors, which was determined by assessing the percentage of cells with positive fluorescence staining, was approximately 37% in both GEM_sen_ and GEM_res_ cells (Fig. 1b). Collectively, these observations confirmed the successful transfection of ov-mTOR expression vectors.

MTT assay revealed that ov-mTOR transfection increased the viability of both GEM_sen_ and GEM_res_ cells in the absence of drug administration, whereas the addition of Evr reduced cell viability compared to that of non-treated cells in all cases (Fig. 1c). We then evaluated glucose uptake (Fig. 1d), ATP production (Fig. 1e), and lactic acid generation (Fig. 1f) in GEM_sen_ and GEM_res_ cells, with or without Evr treatment. We observed an increase in all of the abovementioned parameters upon ov-mTOR transfection in both cell types, while Evr significantly suppressed them in all cases. Next, the expression of proteins associated with aerobic glycolysis was evaluated (Fig. 1g). In GEM_sen_ cells, GLUT1 (Fig. 1h), LDHB (Fig. 1i), HK2 (Fig. 1j), and PKM2 (Fig. 1k) were significantly upregulated by ov-mTOR but downregulated by Evr. In ov-mTOR-transfected GEM_sen_ cells, Evr downregulated these proteins, but the effect was not as prominent as that in cells treated with only Evr. In all cases, GEM_res_ cells exhibited a similar effect, but the overall level of protein expression was higher than that in GEM_sen_ cells, indicating that the effect of aerobic glycolysis was stronger in GEM_res_ cells compared to that in GEM_sen_ cells. However, the suppressive effect of Evr on the ov-mTOR-induced expression of GLUT1 and LDHB in GEM_res_ cells was weaker than that in GEM_sen_ cells. Conversely, the suppressive effect of Evr on the ov-mTOR-induced expression of HK2 and PKM2 was stronger in GEM_res_ cells than that in GEM_sen_ cells.

### Effect of mTOR overexpression and Evr treatment on GEM_sen_ and GEM_res_ cell migration and apoptosis

**Fig. 2:**
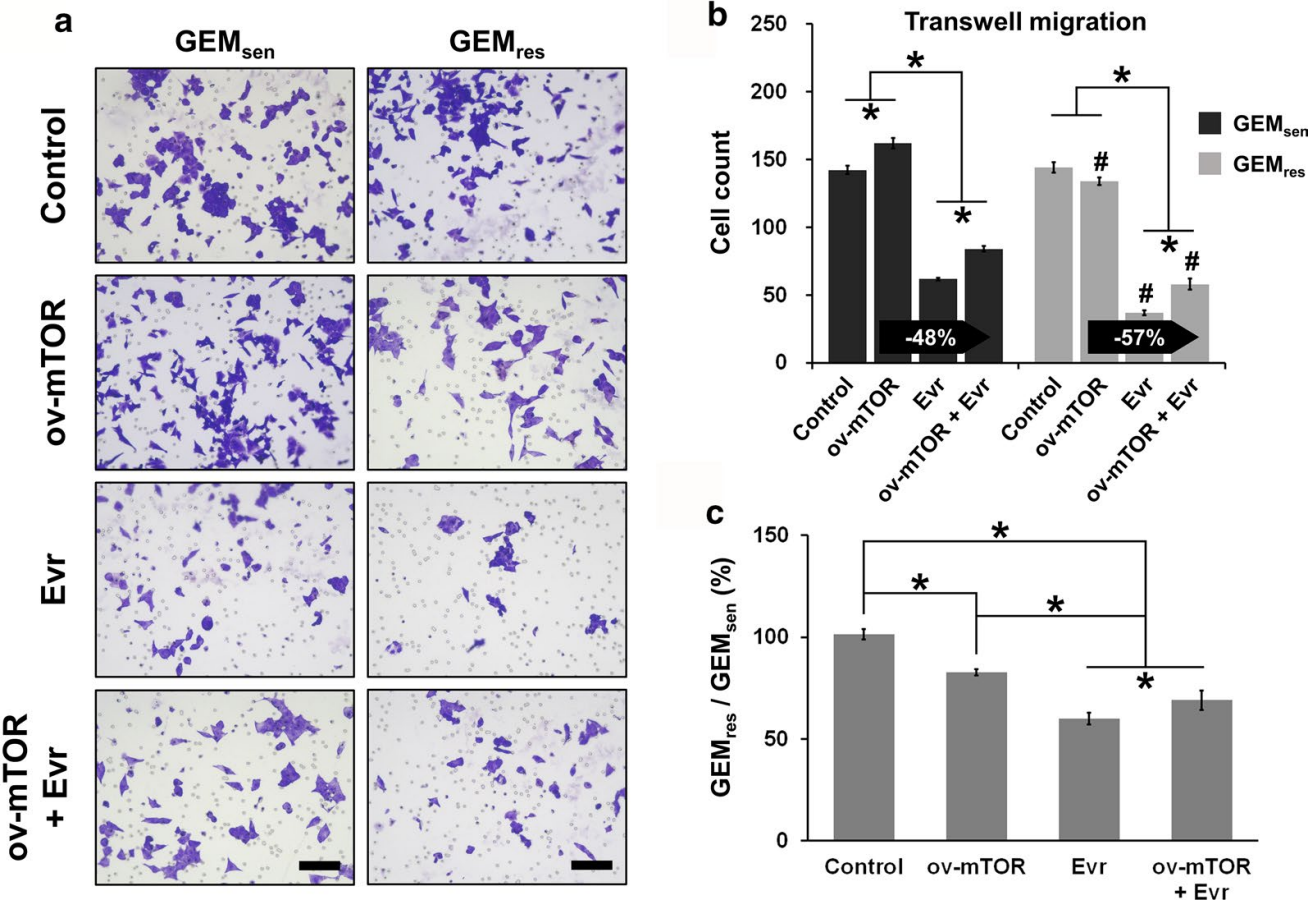
Effect of mTOR overexpression and Evr treatment on GEM_sen_ and GEM_res_ cell migration. **a** Transwell assay of the migration of GEM_sen_ and GEM_res_ cells with or without ov-mTOR transfection or/and Evr treatment after 48 h of incubation. Scale bar, 100 μm. **b** Quantification of the number of migrating GEM_sen_ and GEM_res_ cells. The numbers in the arrows represent the difference (increase or decrease) in cell count between ov-mTOR and ov-mTOR + Evr. In ov-mTOR-transfected GEM_res_ cells, the suppressive effect of Evr on migration ability was stronger than that in ov-mTOR-transfected GEM_sen_ cells. **c** Ratio of the number of migrated GEM_res_ cells to that of migrated GEM_sen_ cells. The data are expressed as the mean ± standard deviation of three replicates (n = 3). *P < 0.05; ^#^P < 0.05 compared with the same treatment in GEM_sen_ cells. GEM: gemcitabine, Evr: everolimus; GEM_sen_: GEM-sensitive pancreatic cancer cells; GEM_res_: GEM-resistant pancreatic cancer cells

We next performed functional analysis to examine the effect of ov-mTOR or/and Evr on the behavior of GEM_sen_ and GEM_res_ cells. Transwell assay (Fig. 2a) revealed that GEM_sen_ cells experienced a significant increase in migration ability after ov-mTOR transfection but a significant decrease with Evr treatment. However, GEM_res_ showed no difference in migration ability after ov-mTOR transfection, while Evr treatment induced a significant decrease. The combined effect of ov-mTOR and Evr was greater than that of ov-mTOR alone but not as significant as Evr alone in both GEM_sen_ and GEM_res_ cells. Importantly, Evr exerted a stronger suppressive effect on migration ability in GEM_res_ cells than that in GEM_sen_ cells after ov-mTOR transfection (Fig. 2b). Comparing the degree of migration between GEM_sen_ and GEM_res_ cells (Fig. 2c), we noticed that the ratio of Evr-treated GEM_res_/GEM_sen_ cells, either with or without ov-mTOR transfection, was lower than that of cells that were not treated by Evr, indicating that Evr had a greater effect in GEM_res_ cells than it did in GEM_sen_ cells in terms of migration suppression.

**Fig. 3:**
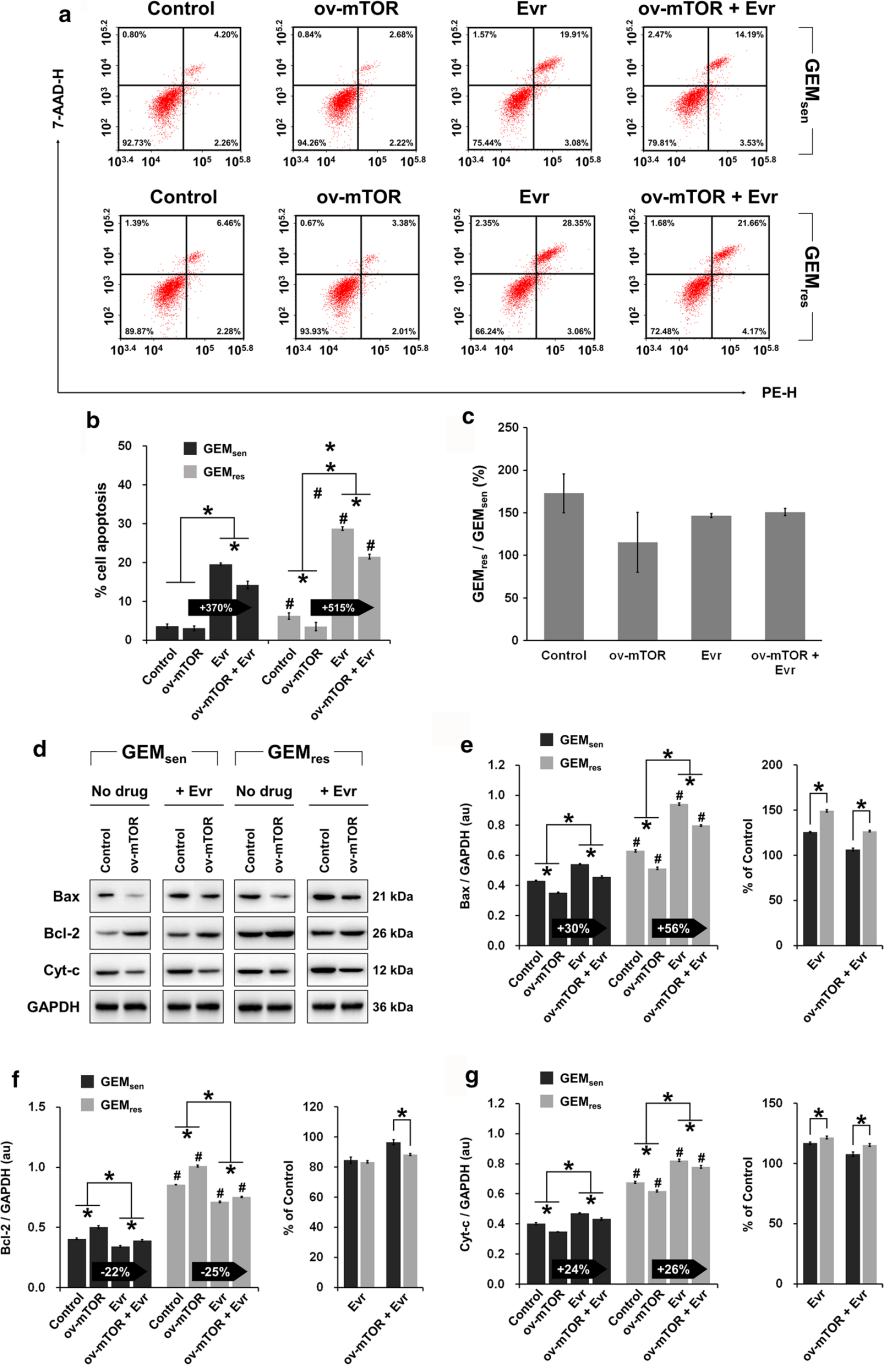
Effect of mTOR overexpression and Evr treatment on the apoptosis of GEM_sen_ and GEM_res_ cells. **a** Flow cytometry of apoptosis in GEM_sen_ and GEM_res_ cells with or without ov-mTOR transfection or/and Evr treatment. Numbers in the upper right quadrant denote the percentage of cells in late apoptosis. **b** Quantification of the proportion of late-apoptotic cells. The numbers in the arrows represent the difference (increase or decrease) in the percentage of late apoptosis between ov-mTOR and ov-mTOR + Evr. In ov-mTOR-transfected GEM_res_ cells, the enhancing effect of Evr on apoptosis was stronger than that in ov-mTOR-transfected GEM_sen_ cells. **c** Ratio of the apoptosis of GEM_res_ cells to that of GEM_sen_ cells. **d** Western blot of proteins associated with apoptosis. Quantification of the protein expression of **e** Bax, **f** Bcl-2, and **g** Cyt-c in GEM_sen_ and GEM_res_ cells with or without ov-mTOR transfection or/and Evr treatment, normalized to that of GAPDH as an internal control. From **e** to **g**, the numbers in the arrows represent the difference (increase or decrease) in protein expression between ov-mTOR and ov-mTOR + Evr. In ov-mTOR-transfected GEM_res_ cells, the overall enhancing effect of Evr on apoptosis was stronger than that in ov-mTOR-transfected GEM_sen_ cells. The data are expressed as the mean ± standard deviation of three replicates (n = 3). *P < 0.05; ^#^P < 0.05 compared with the same treatment in GEM_sen_ cells. GEM: gemcitabine, Evr: everolimus; GEM_sen_: GEM-sensitive pancreatic cancer cells; GEM_res_: GEM-resistant pancreatic cancer cells; au: arbitrary units

In terms of apoptosis (Fig. 3a), a similar trend was observed, wherein ov-mTOR or Evr alone significantly affected GEM_sen_ and GEM_res_ apoptosis, and the combined effect was in between the individual effects. Evr exerted a dramatically stronger enhancing effect on apoptosis in GEM_res_ cells than that in GEM_sen_ cells after ov-mTOR transfection (Fig. 3b). However, comparing the relative degree of apoptosis between GEM_sen_ and GEM_res_ cells by measuring their ratio, we observed that GEM_res_/GEM_sen_ was greater than 100% in all cases (Fig. 3c). This result seems to indicate that apoptosis was more pronounced in GEM_res_ cells, though the difference was not significant between treatments. The expression of apoptosis-related proteins was then examined (Fig. 3d), and the results are consistent with those of flow cytometry (expression profiles of Bax, Bcl-2, and Cyt-c are shown in Fig. 3e–g, respectively). Notably, comparison of protein expression relative to the control group indicated that Evr had a greater pro-apoptotic effect on GEM_res_ cells than it did in GEM_sen_ cells, in both non-transfected and ov-mTOR-transfected cells.

### Effect of mTOR overexpression and Evr treatment on PI3K/AKT/mTOR signaling in GEM_sen_ and GEM_res_ cells

**Fig. 4:**
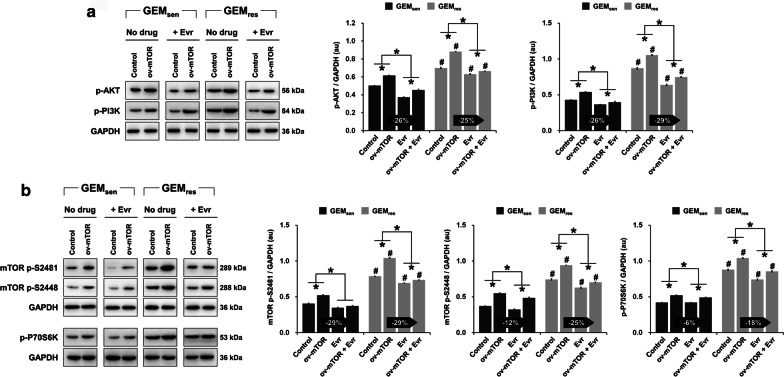
Effect of mTOR overexpression and Evr treatment on PI3K/AKT/mTOR signaling in GEM_sen_ and GEM_res_ cells. **a** Western blot and quantification of the phosphorylation level of AKT and PI3K, normalized to that of GAPDH as an internal control. **b** Western blot and quantification of the phosphorylation level of mTOR (at S2481 and S2448) and P70S6K. The numbers in the arrows represent the difference (increase or decrease) in protein expression between ov-mTOR and ov-mTOR + Evr. The effect of Evr on the phosphorylation of AKT and mTOR (S2481) was similar between ov-mTOR-transfected GEM_sen_ and GEM_res_ cells. However, Evr exerted an overall stronger suppressive effect on the phosphorylation of PI3K, mTOR (S2448), and P70S6K in ov-mTOR-transfected GEM_res_ cells than that in ov-mTOR-transfected GEM_sen_ cells. The data are expressed as the mean ± standard deviation of three replicates (n = 3). *P < 0.05; ^#^P < 0.05 compared with the same treatment in GEM_sen_ cells. GEM: gemcitabine, Evr: everolimus; GEM_sen_: GEM-sensitive pancreatic cancer cells; GEM_res_: GEM-resistant pancreatic cancer cells; au: arbitrary units

Next, we took a brief look at the expression of multidrug resistance-related proteins in GEM_sen_ and GEM_res_ cells (Additional file 1: Supplementary Figure S2). Evidently, ov-mTOR caused a significant increase in the expression of both MDR1 and MRP1 while significantly inhibiting that of BCRP, whereas Evr alone induced the opposite effect in both GEM_sen_ cells. In all cases, the expression of MDR1, MRP1, and BCRP was higher in GEM_res_ cells than that in GEM_sen_ cells, which was anticipated as globally drug-resistant cells express more ATP-binding cassette transporters than do drug-sensitive cells. As we were interested in the potential effect of Evr and GEM on PI3K/AKT/mTOR signaling, we evaluated the phosphorylation levels of components in the signaling pathway. In terms of AKT and PI3K (Fig. 4a), ov-mTOR significantly promoted their phosphorylation in GEM_sen_ cells, while Evr suppressed it. Similarly, ov-mTOR increased the phosphorylation of mTOR at S2481 and S2448 as well as that of P70S6K, but Evr suppressed it in GEM_sen_ cells (Fig. 4b). In all cases, the combined effect of ov-mTOR and Evr in GEM_sen_ was in between those of ov-mTOR and Evr alone, and these effects were observed in parallel in GEM_res_ cells. Furthermore, the effect of Evr on the phosphorylation of AKT was almost the same in ov-mTOR-transfected GEM_sen_ and GEM_res_ cells. However, Evr exerted a stronger suppressive effect on the phosphorylation of PI3K, mTOR (at S2481 and S2448), and P70S6K in GEM_res_ cells than it did on GEM_sen_ cells with ov-mTOR transfection.

### Combined effect of GEM and Evr on tube formation, migration, and apoptosis in GEM_sen_ and GEM_res_ cells

**Fig. 5:**
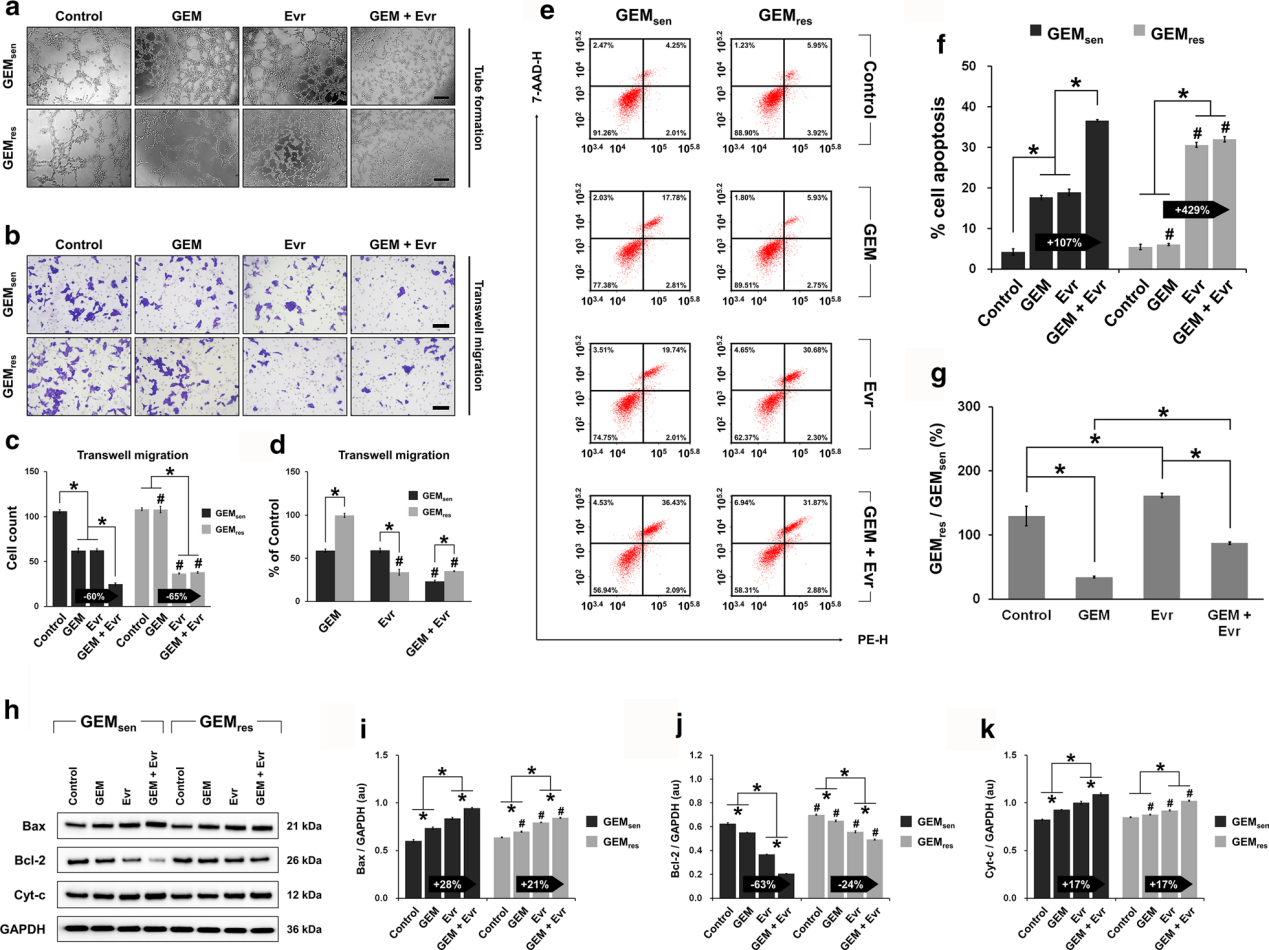
Individual and combined effect of GEM and Evr on tube formation, migration, and apoptosis in GEM_sen_ and GEM_res_ cells. **a** Effect of GEM or/and Evr on tube formation in GEM_res_ and GEM_sen_ cells. Scale bar, 100 μm. **b** Transwell assay and quantification of the migration of GEM_sen_ and GEM_res_ cells treated with GEM or/and Evr after 48 h of culture. Scale bar, 100 μm. **c** Quantification of the number of migrating GEM_sen_ and GEM_res_ cells. The numbers in the arrows represent the difference (increase or decrease) in cell count between GEM and GEM + Evr. In GEM-treated GEM_res_ cells, the enhancing effect of Evr on migration ability was stronger than that in GEM-treated GEM_sen_ cells. **d** Ratio of the number of migrated GEM_res_ cells to that of migrated GEM_sen_ cells. **e** Flow cytometry of apoptosis in GEM_sen_ and GEM_res_ cells treated with GEM or/and Evr. Numbers in the upper right quadrant denote the percentage of cells in late apoptosis. **f** Quantification of the proportion of late-apoptotic cells. The numbers in the arrows represent the difference (increase or decrease) in the percentage of late apoptosis between GEM and GEM + Evr. In GEM-treated GEM_res_ cells, the enhancing effect of Evr on apoptosis was stronger than that in GEM-treated GEM_sen_ cells. **g** Ratio of the apoptosis of GEM_res_ cells to that of GEM_sen_ cells. **h** Western blot of proteins associated with apoptosis. Quantification of the protein expression of **i** Bax, **j** Bcl-2, and **k** Cyt-c in GEM_sen_ and GEM_res_ cells treated with GEM or/and Evr, normalized to that of GAPDH as an internal control. From **i** to **k**, the numbers in the arrows represent the difference (increase or decrease) in protein expression between GEM and GEM + Evr. Compared to GEM-treated GEM_sen_ cells, the effect of Evr on Bax and Bcl-2 expression was weaker in GEM-treated GEM_res_ cells, but Evr exerted similar effect on Cyt-c expression. The data are expressed as the mean ± standard deviation of three replicates (n = 3). *P < 0.05; ^#^P < 0.05 compared with the same treatment in GEM_sen_ cells. GEM: gemcitabine, Evr: everolimus; GEM_sen_: GEM-sensitive pancreatic cancer cells; GEM_res_: GEM-resistant pancreatic cancer cells; au: arbitrary units

We next performed a series of experiments to verify if Evr enhances the sensitivity of pancreatic cancer cells to GEM as speculated. First, the combined effect of GEM and Evr was greater than those of GEM or Evr alone in disrupting tube formation in GEM_sen_ and GEM_res_ cells (Fig. 5a), but this inhibitory effect appeared to be stronger in GEM_res_ cells. Transwell assay (Fig. 5b) demonstrated that individually, GEM and Evr had similar inhibitory effects on GEM_sen_ migration, but the combined effect was the greatest. In GEM_res_ cells, while GEM did not affect migration, Evr significantly inhibited it. However, the combined effect of GEM and Evr was similar to that of Evr alone (Fig. 5c), suggesting that GEM did not exert much therapeutic effect in GEM_res_ cells even in the presence of Evr. Comparing the relative migration of GEM_sen_ and GEM_res_ cells (Fig. 5d), it seems that GEM inhibited GEM_sen_ cell migration to a much greater degree than it did GEM_res_ cell migration, which was expected. On the other hand, the inhibitory effect of Evr on GEM_res_ cell migration was greater than that on GEM_sen_ cell migration. Combining GEM and Evr, migration was inhibited in GEM_sen_ to a significantly higher degree than that induced by GEM alone, but the difference compared to the effect of Evr alone was not significant in GEM_res_ cells.

The results of cell apoptosis (Fig. 5e) corroborate those of cell migration. Notably, the combined administration of GEM and Evr resulted in drastically enhanced GEM_res_ cell apoptosis compared to that in GEM_sen_ cells, relative to GEM only (Fig. 5f). However, this is because combined drug administration had a much greater effect than individual administration in GEM_sen_ cells, whereas GEM_res_ cells were significantly more sensitive to Evr than they were to GEM such that the apoptotic effect was almost solely a result of Evr treatment. Moreover, it appears that GEM_res_ cells were more sensitive to Evr than were GEM_sen_ cells (Fig. 5g). These observations can be complemented with the western blot analysis of specific proteins involved in apoptosis (Fig. 5h). We note here that compared to GEM only, the combination of GEM and Evr induced a greater upregulation of the pro-apoptotic Bax (Fig. 5i) and a greater downregulation of the anti-apoptotic Bcl-2 (Fig. 5j) in GEM_sen_ cells than in GEM_res_ cells, whereas no difference was observed in terms of Cyt-c expression (Fig. 5k). Again, GEM was unable to remarkably induce further apoptosis in Evr-treated GEM_res_ cells because of the inherent GEM resistance of these cells. Even though the results of western blot suggest that combination therapy in GEM_res_ cells was more effective than individual GEM or Evr administration, the ultimate effect was not strong enough to induce meaningful apoptosis of GEM_res_ cells compared to that of GEM_sen_ cells.

### Combined effect of GEM and Evr on glucose metabolism and PI3K/AKT/mTOR signaling in GEM_sen_ and GEM_res_ cells

**Fig. 6:**
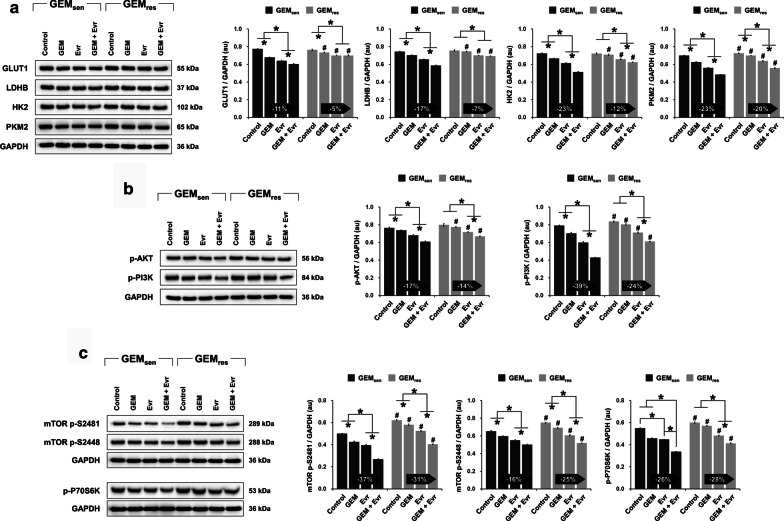
Individual and combined effect of GEM and Evr on PI3K/AKT/mTOR signaling in GEM_sen_ and GEM_res_ cells. Western blot and quantification of **a** proteins associated with the Warburg effect (GLUT1, LDHB, HK2, and PKM2), **b** the phosphorylation level of AKT and PI3K, and **c** the phosphorylation level of mTOR (at S2481 and S2448) and P70S6K in GEM_sen_ and GEM_res_ cells treated with GEM or/and Evr. All protein expression was normalized to that of GAPDH as an internal control. The numbers in the arrows represent the difference (increase or decrease) in protein expression between GEM and GEM + Evr. In GEM-treated GEM_res_ cells, the overall suppressive effect of Evr on the Warburg effect was weaker than that in GEM-treated GEM_sen_ cells. Similarly, in GEM-treated GEM_res_ cells, the suppressive effect of Evr on the phosphorylation of AKT, PI3K, and mTOR (S2481) was weaker than that in GEM-treated GEM_sen_ cells. Conversely, the suppressive effect of Evr on the phosphorylation of mTOR (S2448) and P70S6K was stronger in GEM-treated GEM_res_ cells than that in GEM-treated GEM_sen_ cells. The data are expressed as the mean ± standard deviation of three replicates (n = 3). *P < 0.05; ^#^P < 0.05 compared with the same treatment in GEM_sen_ cells. GEM: gemcitabine, Evr: everolimus; GEM_sen_: GEM-sensitive pancreatic cancer cells; GEM_res_: GEM-resistant pancreatic cancer cells; au: arbitrary units

In terms of glucose metabolism (Fig. 6a), GEM and Evr individually suppressed the protein expression of GLUT1, LDHB, HK2, and PKM2 in GEM_sen_ cells, and their combination exerted a superior effect compared to individual administration. In GEM_res_ cells, GEM only downregulated the expression of GLUT1 and PKM2 but had no effect on LDHB and HK2 expression. Evr, on the other hand, suppressed the expression of all four metabolism-related proteins. When combined, GEM and Evr further suppressed the expression of HK2 and PKM2 compared to Evr alone, but appeared to have no effect on GLUT1 and LDHB. Overall, the suppressive effect of GEM and Evr on the Warburg effect was weaker than that in GEM-treated GEM_res_ cells. Similar observations were noted in terms of the phosphorylation of AKT, PI3K, mTOR (S2481 and 2481), and P70S6K in GEM_sen_ cells, where the suppressive effect of combined GEM and Evr administration was greater than their individual effects (Fig. 6b and c). In GEM-treated GEM_res_ cells, the phosphorylation of AKT, PI3K, and P70S6K remained unchanged, whereas that of mTOR at both S2481 and S2448 was inhibited. Evr, however, was able to suppress the phosphorylation of AKT, PI3K, mTOR (at both S2481 and S2448), and P70S6K in GEM_res_ cells. The suppressive effect of Evr on the phosphorylation of AKT, PI3K, and mTOR (S2481) was weaker than that in GEM-treated GEM_sen_ cells. Conversely, the suppressive effect of Evr on the phosphorylation of mTOR (S2448) and P70S6K was stronger in GEM-treated GEM_res_ cells than that in GEM-treated GEM_res_ cells. It is also interesting to note that while GEM did not affect the phosphorylation of AKT, PI3K, and P70S6K in GEM_res_ cells, when combined with Evr, GEM did contribute to a further suppressive effect on the phosphorylation of these factors.

### In vivo evaluation of the combined effect of GEM and Evr on pancreatic tumor progression

**Fig. 7:**
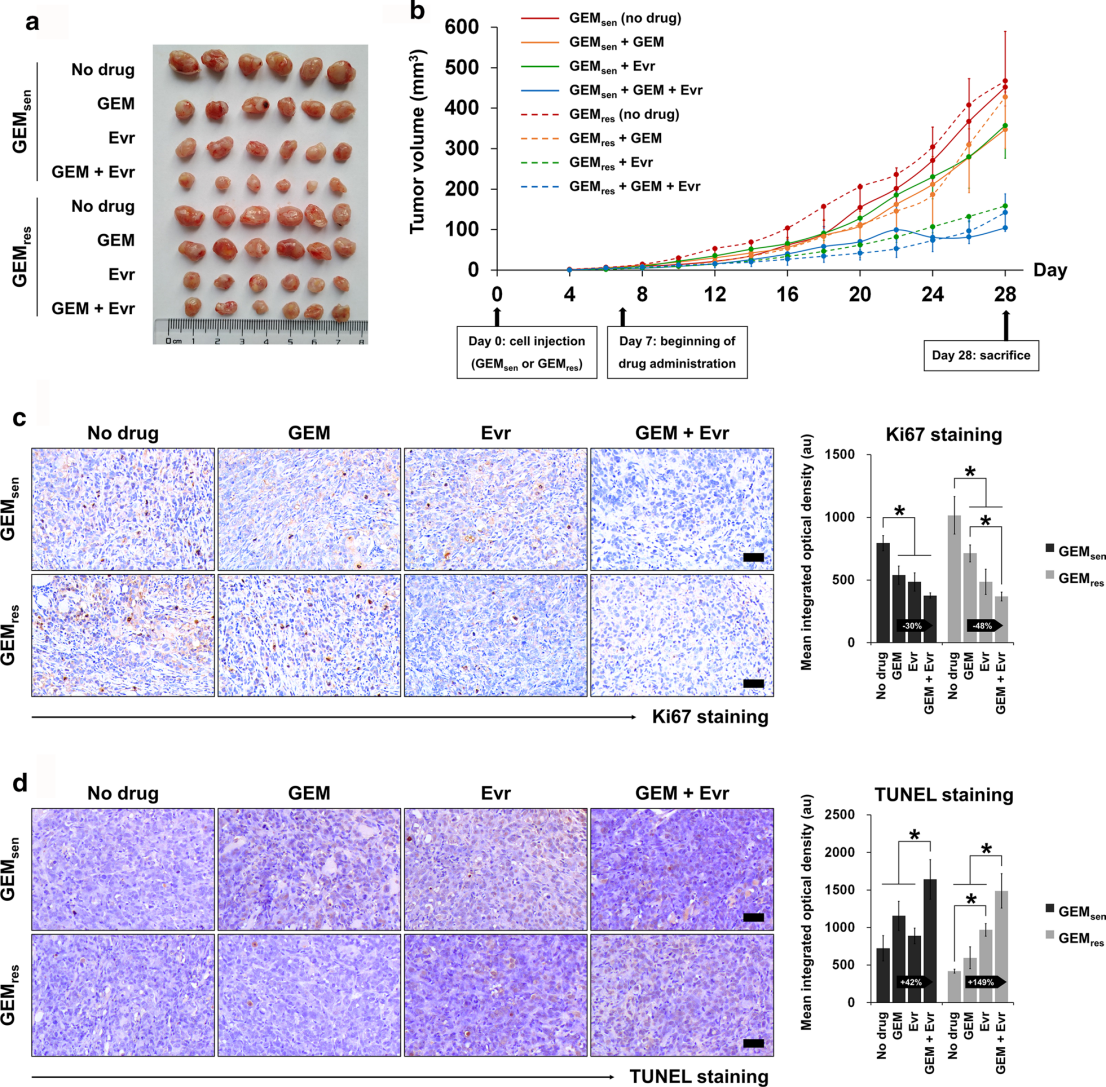
In vivo evaluation of the individual and combined effect of GEM and Evr on pancreatic tumor progression. Six-week-old BALB/C nude mice were subcutaneously injected with GEM_sen_ or GEM_res_ cells and administered GEM or/and Evr after one week of tumor formation. **a** Macroscopic observation of tumors extracted from xenografted animals at the end of the 28-day experimental period. **b** Tumor growth curve showing the change in tumor volume over the experimental period. Tumor volume was measured every two days. GEM_sen_ and GEM_res_ tumors were denoted by solid and dashed lines, respectively. **c** Ki67 staining and quantification of tissue proliferation in GEM_sen_- or GEM_res_-transplanted tumors treated with GEM or/and Evr. Scale bar, 50 μm. **d** TUNEL staining and quantification of tissue apoptosis in GEM_sen_- or GEM_res_-transplanted tumors treated with GEM or/and Evr. Scale bar, 50 μm. The relative amount of Ki67-positive and TUNEL-positive staining was evaluated using ImagePro Plus and expressed as the mean integrated optical density of brown staining. The numbers in the arrows represent the difference (increase or decrease) in positive staining between GEM and GEM + Evr. In GEM-treated GEM_res_ tumors, the suppressive effect of Evr on tissue proliferation and the enhancing effect of Evr on tissue apoptosis were stronger than those in GEM-treated GEM_sen_ tumors. The data are expressed as the mean ± standard deviation of six replicates (n = 6). *P < 0.05; ^#^P < 0.05 compared with the same treatment in GEM_sen_ tumors. GEM: gemcitabine, Evr: everolimus; GEM_sen_: GEM-sensitive pancreatic cancer tumors; GEM_res_: GEM-resistant pancreatic cancer tumors; TUNEL: terminal deoxynucleotidyl transferase dUTP nick end labeling; au: arbitrary units

Finally, the combined effect of GEM and Evr on pancreatic cancer was evaluated in an in vivo model of xenografted tumors. As demonstrated in Fig. 7a and b, GEM alone did not seem to have an effect on GEM_res_-transplanted tumors and in fact, the tumor volume was higher than that of the GEM-treated GEM_sen_-transplanted tumors at the end of the experimental period. Remarkably, the administration of Evr alone suppressed the growth of GEM_res_-transplanted tumors drastically compared to that of GEM_sen_-transplanted tumors. The combined administration of GEM and Evr evidently inhibited the progression of GEM_sen_-transplanted tumors compared to the effect of GEM or Evr alone by the end of the experimental period. In GEM_res_-transplanted tumors, this seemed to be mainly attributed to the therapeutic action of Evr, as was the case in vitro, and GEM had little influence on GEM_res_ tumor growth even in the presence of Evr. Ki67 (Fig. 7c) and TUNEL staining (Fig. 7d) were performed to evaluate tumor cell proliferation and apoptosis, respectively. GEM_sen_-transplanted tumor cell proliferation was inhibited by individual administration of GEM or Evr to similar degrees, but their combination further enhanced this inhibition. This effect was similarly observed in GEM_res_-transplanted tumors but critically, the combination of GEM and Evr suppressed GEM_res_-transplanted tumor proliferation to a much higher degree than it did in GEM_sen_-transplanted tumors, compared to GEM alone (% change in Fig. 7C). Parallel results were observed in terms of tumor tissue apoptosis, where the combination of GEM with Evr inhibited tumor growth by promoting apoptosis in GEM_res_-transplanted tumor tissues to a much greater degree than it did in GEM_sen_-transplanted tissues, compared to GEM alone (% change in Fig. 7d). Consistent with the in vitro findings, the effect of Evr on tumor cell proliferation and apoptosis appeared to be stronger in GEM_res_-transplanted tumors than that in GEM_sen_-transplanted tumors.

## Discussion

This study investigated the processes underlying the development of GEM resistance in pancreatic cancer cells and the effect of the mTOR inhibitor Evr therein. To do this, mTOR was overexpressed in GEM_sen_ and GEM_res_ pancreatic cancer cells and Evr (alone or in conjunction with GEM) was used as a therapeutic agent to treat these cells. We demonstrated that Evr exhibited anti-cancer properties by inhibiting the activation of the PI3K/AKT/mTOR pathway to regulate glucose metabolism, tube formation, cell migration, and apoptosis in pancreatic cancer cells. Overall, the effect of Evr was stronger in GEM_res_ cells and tumors than that in GEM_sen_ cells and tumors, but the current results are insufficient to conclusively infer that Evr had significant enhancing effects on the sensitivity of GEM_res_ cells to GEM.

Although GEM has led to important progress in pancreatic cancer treatment in the past decade, its efficacy is limited. To address the problem of GEM resistance, it is important to elucidate the underlying mechanisms involved in its occurrence. Malignant transformation of normal cells is accompanied by aerobic glycolysis, which leads to increased glucose uptake and lactic acid production (Warburg 1956). The metabolic reactions of aerobic glycolysis are catalyzed by enzymes such as HK2, LDHB, and PKM2, which are implicated in cancer (Vander Heiden 2010; Cui 2015). Cellular glucose uptake is promoted by a family of 14 transporters known as GLUTs, of which GLUT1, GLUT3, and GLUT4 are the most widely studied in cancer (Thorens and Mueckler 2010). Elevated expression of GLUT1, which is consequently involved in the activation of mTOR, has been shown in numerous tumors and leads to an increase in glycolysis (Wang et al. 2013; Bhattacharya 2014). In this study, the protein levels of GLUT1, LDHB, HK2, and PKM2 in GEM_sen_ and GEM_res_ cells were significantly decreased by Evr through mTOR inhibition. Meanwhile, glucose uptake, lactic acid levels, and ATP production were suppressed by Evr. These observations indicated that glycolysis was inhibited by suppressing mTOR activation in pancreatic cancer cells. In a related manner, the occurrence of the Warburg effect could affect drug efficacy, as increased levels of LDH were shown to result in resistance to trastuzumab in breast cancer (Zhao 2011). We thus speculated that targeting the Warburg effect using Evr might be beneficial in combatting the resistance of pancreatic cancer cells to GEM.

The results of our study demonstrated that overexpression of mTOR activated the Warburg effect in GEM_sen_ and GEM_res_ cells, implicating that mTOR may be a factor regulating GEM resistance via PI3K/AKT/mTOR signaling. The PI3K/AKT pathway is involved in oncogenesis in many types of malignancies (Dey et al. 2017) and is constitutively activated in most human pancreatic cancer cell lines. The combination of a PI3K inhibitor with GEM has demonstrated potential therapeutic effect against pancreatic cancer (Ng et al. 2000; Bondar et al. 2002). As a downstream protein of PI3K/AKT, mTOR plays an important role in cell proliferation and survival (Blenis 2017). Studies have revealed that mTOR was activated in pancreatic cancer tissues, and mTOR inhibitors are currently applied in molecular-targeted cancer therapies in clinical treatment (Vignot et al. 2005; Kagawa 2012). While phosphorylation of mTOR complex 1 and 2 occur at S2448 and S2481, respectively (Seo 2018; Watanabe et al. 2011), mTOR itself phosphorylates multiple substrate proteins including P70S6 (Tavares 2015; Qin et al. 2016). It has been shown that downregulation of mTOR and P70S6K phosphorylation is associated with the inhibition of pancreatic tumor proliferation and metastasis (Zheng 2020), which is in agreement with the results shown in our study through the use of GEM and/or Evr.

Evr is an inhibitor of mTOR that has exhibited anti-cancer efficacy in several cancers via the regulation of PI3K/AKT/mTOR signaling (Juengel 2017; Du 2018). Previous reports have shown evidence of the combined anti-tumor effects of mTOR inhibition and GEM in pancreatic cancer (Okada et al. 2007; Ito 2006). Our findings indicate that Evr was able to reverse the effects of mTOR overexpression on glucose metabolism, migration capability, apoptosis, and PI3K/AKT/mTOR signaling in GEM_sen_ and GEM_res_ cells. Furthermore, while GEM alone showed little to no effect in GEM_res_ cells, Evr significantly weakened the Warburg effect in GEM_res_ cells while inhibiting their migration and promoting their apoptosis.

We initially hypothesized, based on available evidence in the literature, that Evr would enhance the sensitivity of pancreatic cancer cells (especially GEM_res_ cells) to GEM, but comprehensive analysis of our results does not fully support this conjecture. However, we identified several individual proteins, the expression of which was not affected by GEM in GEM_res_ cells but showed significant downregulation with combined GEM and Evr treatment (compared to their effects when administered alone). Namely, the proteins showing such a trend were cytochrome-c, LDHB, HK2, and p-P70S6K. Cytochrome-c is a critical component involved in the initiation of mitochondrial apoptosis (Odinokova 2009), and its activation was associated with the therapeutic effects of anti-cancer drugs in pancreatic ductal adenocarcinoma (Cheng 2019). Interestingly, LDHB and HK2 are localized to the inner and outer mitochondrial membrane (Cruz-Lopez et al. 2019; Nagdas 2019), respectively, and there may be a link between their expression and mitochondrial apoptosis in cancer cells. In terms of P70S6K, an understanding of its specific role as a substrate of mTOR may provide clues that enable us to evaluate its unique functions in the mechanisms of action of Evr.

Our study is subject to several limitations. The major drawback is that the experimental results only partially supported that Evr promoted the sensitivity of GEM_res_ cells to GEM. Although an increase in GEM sensitivity was not globally achieved by Evr, we speculate that the proteins identified previously (cytochrome-c, LDHB, HK2, and p-P70S6K) are key factors that contribute to Evr-mediated sensitivity to GEM. In-depth study of these factors in conjunction with Evr will be required to further elucidate their individual roles in regulating GEM sensitivity, especially with respect to the possible involvement of mitochondrial dysfunction and apoptosis. In addition, studies of oxygen consumption rate and oxygraphy experiments that reveal the respiration/fermentation ratio of pancreatic cancer cells were not carried out here because of technical constraints. These experiments will be designed for future studies in order to gain deeper insight into the effect of Evr-regulated metabolism on GEM resistance.

## Conclusions

In conclusion, we revealed that Evr effectively impaired the growth of GEM-resistant pancreatic cancer cells, both in vitro and in vivo, by regulating the PI3K/AKT/mTOR signaling pathway. We also suggest that the strong anti-cancer properties of Evr in GEM_res_ cells are closely linked to mTOR-mediated glucose metabolism and the Warburg effect. Taken together, Evr plays an essential role as an mTOR inhibitor and can be considered as an adjuvant or complementary agent to GEM, as its therapeutic effects against GEM-resistant pancreatic cancers have been further clarified. The possible role of mitochondrial apoptosis, as alluded to in the current study, should be thoroughly examined in follow-up studies in order to elucidate the link between Evr and GEM sensitivity. The findings presented here accentuate the clinical value of Evr as a part of therapeutic strategies against pancreatic cancer and warrant continued investigation in this regard.

## Supplementary Information

**Additional file 1.** Supplementary figures.

## Data Availability

The datasets generated for this study are available on request to the corresponding author.
