# Supplementary material for: Everolimus regulates the activity of gemcitabine-resistant pancreatic cancer cells by targeting the Warburg effect via PI3K/AKT/mTOR signaling
Source: Mol Med. 2021 Apr 13;27:38. doi: 10.1186/s10020-021-00300-8 (PMC8045370; doi:10.1186/s10020-021-00300-8)
Supplement: Supplementary file 1 — Additional file 1. Supplementary figures. [file 10020_2021_300_MOESM1_ESM.docx]

Supplementary Material

# Supplementary Figures


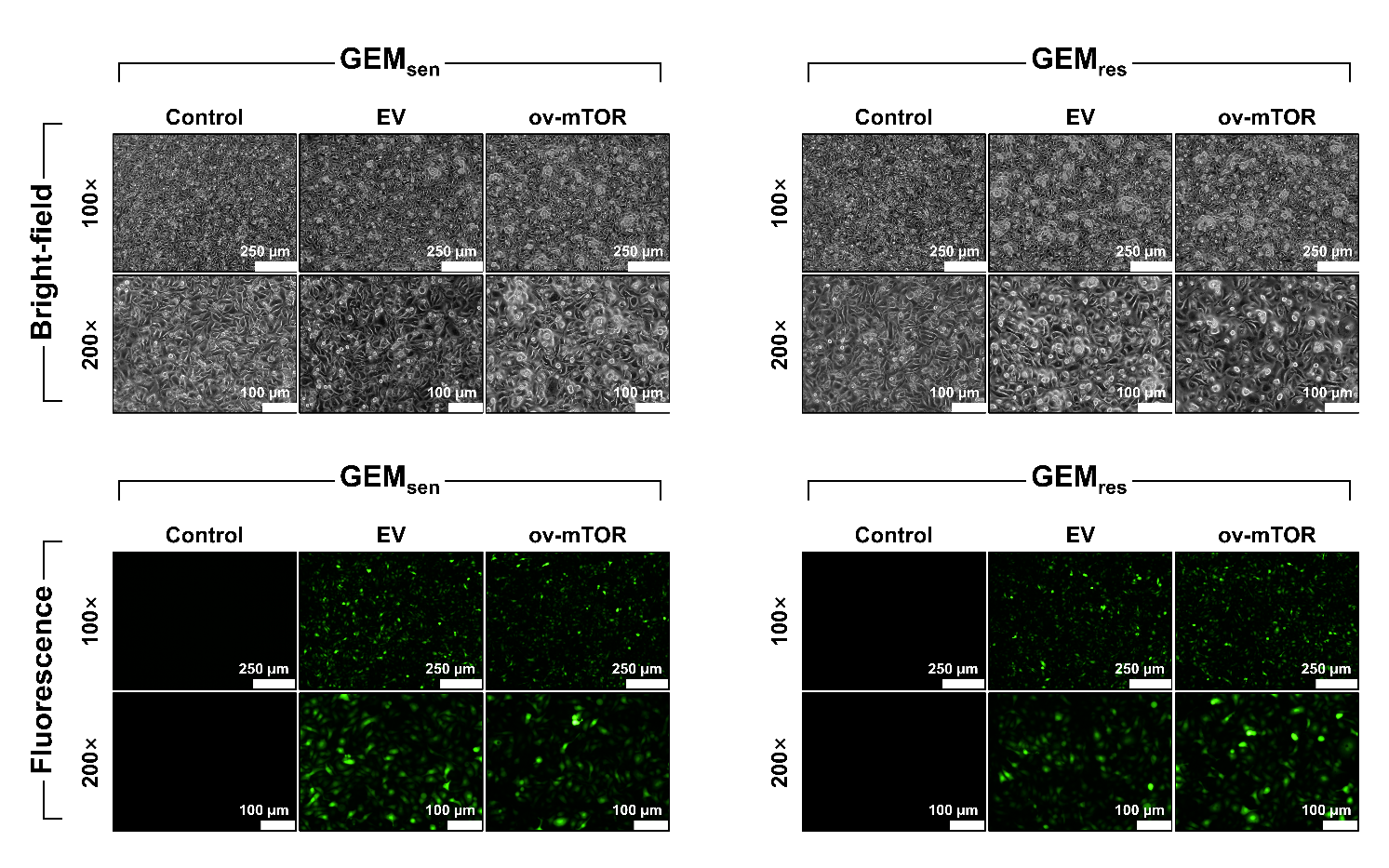


**Supplementary Figure S1. Characterization of transfected GEM_sen_ and GEM_res_ cells.** GEM_sen_ and GEM_res_ cells were either untransfected (Control) or transfected with empty fluorescent vectors (EV) or mTOR overexpression vectors (ov-mTOR). The morphology of the cells was visualized using bright-field microscopy at 100× and 200× and the transfection efficiency was evaluated by examining the green fluorescence expressed by the transfected vectors using fluorescence microscopy at 100× and 200×. GEM: gemcitabine, Evr: everolimus; GEM_sen_: GEM-sensitive pancreatic cancer cells; GEM_res_: GEM-resistant pancreatic cancer cells.

**
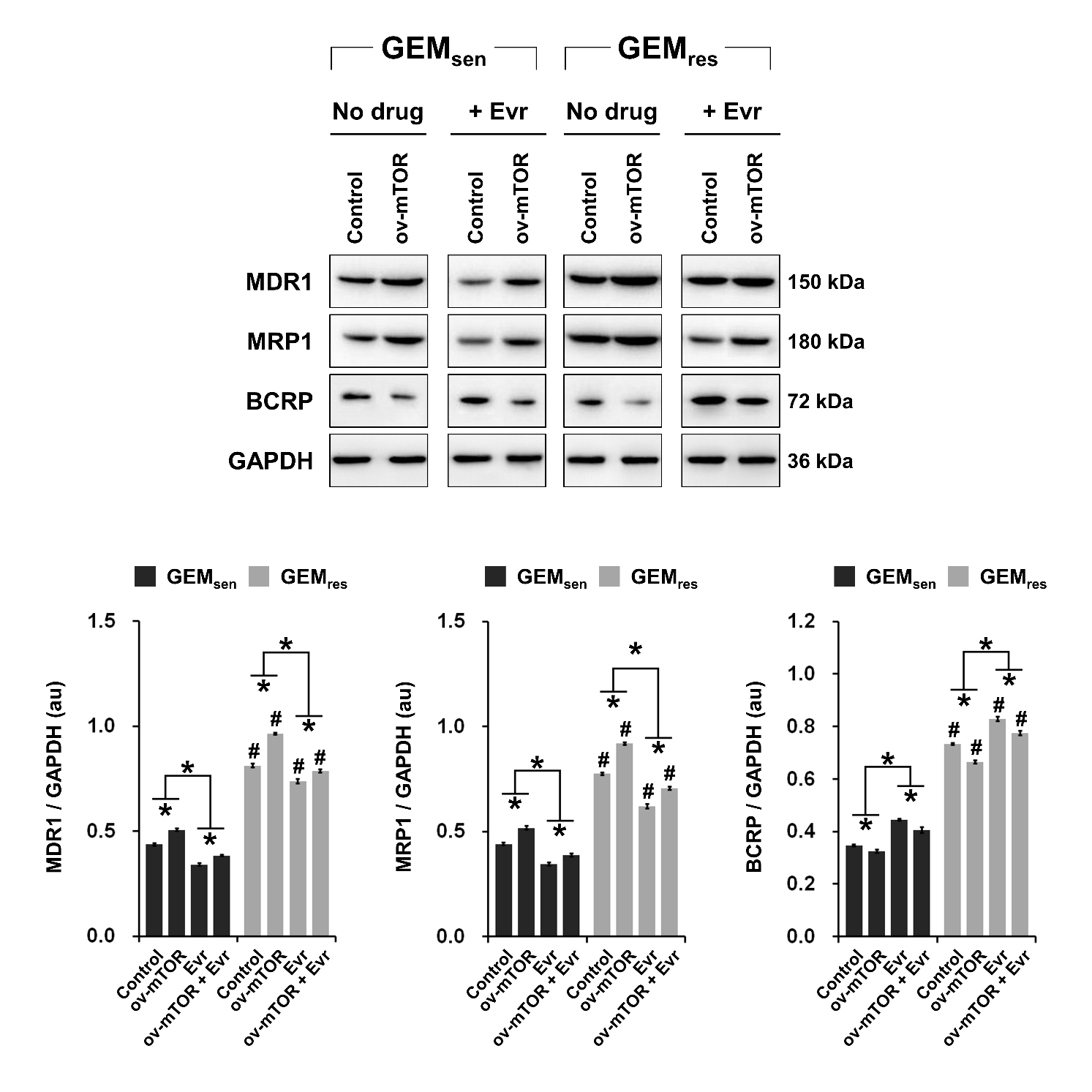
**

**Supplementary Figure S2.** **Effect of mTOR overexpression and Evr treatment on multidrug resistance in GEM_sen_ and GEM_res_ cells.** Western blot and quantification of the protein expression of MDR1, MRP1, and BCRP in GEM_sen_ and GEM_res_ cells with or without ov-mTOR transfection and/or Evr treatment. All protein expression is normalized to that of GAPDH as an internal control. The data are expressed as the mean ± standard deviation of three replicates (n = 3). * denotes P < 0.05; # denotes P < 0.05 compared with the same treatment in GEM_sen_ cells. GEM: gemcitabine, Evr: everolimus; GEM_sen_: GEM-sensitive pancreatic cancer cells; GEM_res_: GEM-resistant pancreatic cancer cells.
